# LSD Increases Primary Process Thinking via Serotonin 2A Receptor Activation

**DOI:** 10.3389/fphar.2017.00814

**Published:** 2017-11-08

**Authors:** Rainer Kraehenmann, Dan Pokorny, Helena Aicher, Katrin H. Preller, Thomas Pokorny, Oliver G. Bosch, Erich Seifritz, Franz X. Vollenweider

**Affiliations:** ^1^Department of Psychiatry, Psychotherapy and Psychosomatics, Psychiatric Hospital, University of Zurich, Zürich, Switzerland; ^2^Neuropsychopharmacology and Brain Imaging Unit, Department of Psychiatry, Psychotherapy and Psychosomatics, Psychiatric Hospital, University of Zurich, Zürich, Switzerland; ^3^Department of Psychosomatic Medicine and Psychotherapy, University of Ulm, Ulm, Germany

**Keywords:** LSD, ketanserin, 5-HT2A receptor, mental imagery, primary and secondary process thinking, primary emotions, cognitive bizarreness, healthy subjects

## Abstract

**Rationale:** Stimulation of serotonin 2A (5-HT2A) receptors by lysergic acid diethylamide (LSD) and related compounds such as psilocybin has previously been shown to increase primary process thinking – an ontologically and evolutionary early, implicit, associative, and automatic mode of thinking which is typically occurring during altered states of consciousness such as dreaming. However, it is still largely unknown whether LSD induces primary process thinking under placebo-controlled, standardized experimental conditions and whether these effects are related to subjective experience and 5-HT2A receptor activation. Therefore, this study aimed to test the hypotheses that LSD increases primary process thinking and that primary process thinking depends on 5-HT2A receptor activation and is related to subjective drug effects.

**Methods:** Twenty-five healthy subjects performed an audio-recorded mental imagery task 7 h after drug administration during three drug conditions: placebo, LSD (100 mcg orally) and LSD together with the 5-HT2A receptor antagonist ketanserin (40 mg orally). The main outcome variable in this study was primary index (PI), a formal measure of primary process thinking in the imagery reports. State of consciousness was evaluated using the Altered State of Consciousness (5D-ASC) rating scale.

**Results:** LSD, compared with placebo, significantly increased primary index (*p* < 0.001, Bonferroni-corrected). The LSD-induced increase in primary index was positively correlated with LSD-induced disembodiment (*p* < 0.05, Bonferroni-corrected), and blissful state (*p* < 0.05, Bonferroni-corrected) on the 5D-ASC. Both LSD-induced increases in primary index and changes in state of consciousness were fully blocked by ketanserin.

**Conclusion:** LSD induces primary process thinking via activation of 5-HT2A receptors and in relation to disembodiment and blissful state. Primary process thinking appears to crucially organize inner experiences during both dreams and psychedelic states of consciousness.

## Introduction

There is now accumulating evidence (Sloman and Steinberg, 1996; Evans, 2008; Shanks, 2010) confirming and extending the early meta-psychological theory of Freud (Pribram and Gill, 1976) which posits that there exist two distinct modes of psychic functioning: primary process and secondary process. It is broadly believed that in normal adults, secondary process is a hierarchically higher-level cognitive mode which fulfills an adaptive, reflective, rule-bound function (“reality principle” in Freudian terms) and thus inhibits lower-level, automatic, motivation- and emotion-driven primary process (“pleasure principle” in Freudian terms) (Arminjon, 2011). Under altered psychophysiological conditions such as dreaming, hypnosis, meditation, sensory deprivation, respiratory maneuvers, trance, psychosis, and epilepsy, primary process may become the prevailing cognitive mode (Barr et al., 1972; Vaitl et al., 2005; Hermle and Kraehenmann, 2017). Primary process thinking can be operationalized and reliably assessed using formal linguistic measures such as image fusion; unlikely combinations or events; sudden shifts or transformations of images; and contradictory or illogical actions, feelings, or thoughts (Rapaport, 1950; Holt, 1956; Auld et al., 1968; Shevrin, 1996; Sloman and Steinberg, 1996; Brakel et al., 2000).

Previous studies (Landon and Fischer, 1970; Martindale and Fischer, 1977; Natale et al., 1978; Spitzer et al., 1996; Family et al., 2016; Kraehenmann et al., 2017) indicate that classical psychedelics such as lysergic acid diethylamide (LSD) and related compounds such as psilocybin activate mental processes which are closely related to primary process, such as vivid, dreamlike imagery, basic emotions, and bizarre thinking. For example, early linguistic studies (Landon and Fischer, 1970; Martindale and Fischer, 1977; Natale et al., 1978) investigated the effects of psychedelics on thought content using primary process dictionaries. They found that psychedelics acutely increase frequency of primary process words in subjective reports of healthy subjects. Moreover, recent studies (Spitzer et al., 1996; Family et al., 2016) showed that psychedelics enhance access to remote and non-obvious associations in tasks where subjects have to rely on automatic, intuitive and uncontrolled thinking. Finally, we (Kraehenmann et al., 2017) recently investigated the effects of LSD on imagery reports of healthy subjects. We found that LSD increased cognitive bizarreness, a formal measure of dreaming cognition, via activation of the serotonin 2A (5-HT2A) receptor. Taken together, previous research on the effects of psychedelics on thought processes indicate that psychedelics may shift cognition toward primary process thinking.

However, it is still largely unknown whether LSD induces primary process thinking under placebo-controlled, standardized experimental conditions, and whether these effects are related to subjective experience and 5-HT2A receptor activation. A better understanding of the cognitive mechanisms underlying psychedelic states of consciousness is important, especially given that there is accumulating qualitative evidence (Gasser et al., 2015; Belser et al., 2017; Watts et al., 2017) indicating that the therapeutic effects of psychedelics may be mediated by their acute effects on subjective experience. Therefore, in this study, we compared the post-peak effects of LSD, placebo and LSD after pre-treatment with the 5-HT2A receptor antagonist ketanserin on primary process thinking in mental imagery reports of healthy subjects. Primary index (PI), a formal measure of primary process thinking (Stigler, 2001; Frick et al., 2008), was used as primary endpoint in this study. We hypothesized that LSD would increase PI in verbal imagery reports. We further hypothesized that ketanserin would block the effects of LSD on PI and subjective experience. Finally, given the relative novelty of the primary endpoint variable (PI) in the field of cognitive neuroscience, we performed multiple correlation analyses to quantitatively assess the relationship between PI and other more common measures which had been frequently used to assess psychedelic-induced changes in state of consciousness, using a short version of the Altered State of Consciousness (5D-ASC) self-rating scale; ratings of mental imagery experience, using visual analog scales (VASs); and dreaming cognition, using cognitive bizarreness (BD) in the mental imagery reports.

## Materials and Methods

### Study Design

The study followed a double-blind, placebo-controlled, within-subjects, crossover design that involved three experimental sessions in balanced order. The washout periods between sessions were at least 14 days. This study was carried out in accordance with the recommendations of the Declaration of Helsinki and International Conference on Harmonization Guidelines in Good Clinical Practice (ICH-GCP). All subjects gave written informed consent. The protocol was approved by the Cantonal Ethics Committee of Zurich. The administration of LSD in healthy subjects was authorized by the Swiss Federal Office for Public Health, Bern, Switzerland.

### Participants

Twenty-five healthy subjects (19 men, 6 women; mean age ± SD: 25 ± 4 years; range: 20–34 years) participated in the study. Subjects had to be physically and mentally healthy. Exclusion criteria were pregnancy, poor knowledge of the German language, history of alcohol or illicit drug dependence, and previous significant adverse reaction to a psychedelic drug. Nine of the 25 subjects had prior experience with classic psychedelics (number of subjects: psilocybin 6, LSD 3, LSA^1^ 1, DMT^2^ 1, 2C-E^3^ 1).

### Study Procedures

The mental imagery task from this study has been described in detail elsewhere (Kraehenmann et al., 2017). Briefly, the 30-min task followed the mental imagery method developed by Leuner (1969) and was performed 7 h after drug treatment, during the descending phase of the acute effects of LSD (Dolder et al., 2015). The task was conducted in an esthetic living-room-like room located in a tranquil side wing of the research department. Mental imagery reports from the subjects were audio recorded and transcribed for statistical analysis.

### Study Drug

In each of the three experimental sessions, subjects first received pre-treatment, followed by treatment after 1 h. The drug conditions were LSD (placebo + 100 mcg LSD orally), Ket+LSD (40 mg ketanserin + 100 mcg LSD orally), and Pla (placebo + placebo orally).

## Measures

### State of Consciousness and Mental Imagery Experience

Subjective state of consciousness at the time of the mental imagery task (390 min after drug intake) was evaluated using a short version of the Altered State of Consciousness (5D-ASC) rating scale (Studerus et al., 2010) for spiritual experience, blissful state, disembodiment, elementary imagery, and changed meaning of percepts. Mental imagery experience was evaluated using visual analog subscales (VASs) for visual vividness, emotional arousal, positive emotions, negative emotions, insight and relaxation.

To assess the relationship between primary process thinking and dream mentation, we included cognitive bizarreness (BD) in the multiple correlations analysis of this study (cognitive bizarreness is a standardized formal measure of dream mentation and had been calculated from the mental imagery reports of this study sample (*N* = 25) elsewhere, see Kraehenmann et al., 2017).

### Primary and Secondary Process Thinking

The main outcome measure in this study was primary index (PI), a formal measure of primary process thinking which had been previously used in text-analytical studies on primary process thinking and mental imagery (Stigler, 2001; Frick et al., 2008). PI was calculated by dividing the relative frequency of primary process (PP) scores by the sum of primary process and secondary process (SP) scores in the imagery reports (PI = 100 × PP/(PP+SP)) (Stigler, 2001). The relative frequency of PP and SP scores was calculated by dividing the PP and SP scores by the number of words in the reports.

Primary process was evaluated using the rating scale of Auld et al. (1968), a comprehensive scale for measuring primary process thinking. The scale consists of nine PP categories (condensation, unlikely combinations or events, fluid transformations, visual representation, symbolism, contradiction, magic occurrences, inhibited movement, taboo sexual and aggressive acts) which sum up to the PP score. Examples for PP items from our study subjects: “…a cat is coming from the right side. The cat has huge blue and luminous eyes…the eyes look upward, then down, left, right, always alternating, like a cuckoo clock with moving eyes…now she has turned into a wooden clock hanging on a wall”; “I am part of a metal plate…I am fusing with the metal plate…I am now a part of this plate…it feels like being a liquid…I am only existing in certain parts of my body…The whole room rolls itself and suddenly, everything is dark…I can only see flickering light and two-dimensional faces”; “I see two entangled persons, like an art painting…when I approach the two persons, they form an ugly bulb and dissolve into bubbles…now I see a huge mouth with yellow teeth…the mouth snaps and draws everything in.”

Secondary process was evaluated using a modified version (Natale et al., 1979) of the rating scale of Weintraub and Aronson (1969), a comprehensive scale for measuring secondary process thinking. The scale consists of seven SP categories (non-personal reference, negators, qualifiers, retractors, explaining, expressions of feeling, evaluators) which sum up to the SP score. Examples for SP items from our study subjects: “…the room has got a bed. The bed is covered with a blanket protecting the bed from dust, I suppose. The bed is adorned with two or three carefully arranged pillows - this looks beautiful…”; “It is a little brook with trees along the banks…actually, the water is really cold…it feels good…the water is indeed cold as ice and it is freezing, but because I only dip my feet in the water, it is an extremely good feeling, vitalizing…”; “…it is not such a special house…it has a roof, a balcony, windows, a garden…in front of the house there is scrub, plants…and a green meadow…the meadow is not so beautiful, doesn’t look quite as well cared for as it should…”.

## Statistics

The statistical analyses were performed using IBM SPSS Statistics 23 software (IBM, Chicago, IL, United States). Repeated-measures analyses of variance (ANOVAs) were conducted to compare the drug effects in LSD, Ket+LSD, and Pla conditions. Significant main effects or interactions in the ANOVAs were followed by Bonferroni-corrected *post hoc* pairwise comparisons with a significance level of *p* < 0.05 (two-tailed test). Bonferroni-corrected Spearman multiple correlations (Bonferroni-corrected alpha = 0.05/12 = 0.0042) were used to quantify the relations between the LSD-Pla difference scores for primary index (ΔPI), state of consciousness (Δ5D-ASC), mental imagery experience (ΔVAS), and cognitive bizarreness (ΔBD).

## Results

### State of Consciousness and Mental Imagery Experience

Lysergic acid diethylamide significantly changed state of consciousness, as indicated by a significant main effect of drug [*F*(2,48) = 89.42, *p* < 0.001, $\eta_{p}^{2}$ = 0.79) in a repeated-measures (drug × subscale) ANOVA on 5D-ASC score at T3. There was also a significant main effect of subscale [*F*(4,96) = 17.63, *p* < 0.001, $\eta_{p}^{2}$ = 0.42] and a significant drug × subscale interaction [*F*(8,192) = 16.01, *p* < 0.001, $\eta_{p}^{2}$ = 0.40]. Bonferroni-corrected *post hoc* pairwise comparisons revealed a greater score on all five 5D-ASC subscales in the LSD condition than in the Pla and Ket+LSD conditions (all *p* < 0.05). Scores did not differ between the Pla and Ket+LSD conditions for any 5D-ASC subscale (all *p* = n.s.), indicating that ketanserin pre-treatment completely blocked all LSD-induced effects (**Supplementary Figure S1**).

Lysergic acid diethylamide significantly changed subjective mental imagery experience, as indicated by a significant main effect of drug [F(2,48) = 8.57, p < 0.001, $\eta_{p}^{2}$ = 0.26] in a repeated-measures (drug × subscale) ANOVA on the retrospectively administered VAS for mental imagery experience. There was also a significant main effect of subscale [F(2.86,68.71) = 55.23, p < 0.001, $\eta_{p}^{2}$ = 0.70] and a significant drug × subscale interaction [F(5.58, 133.86) = 3.21, p = 0.007, $\eta_{p}^{2}$ = 0.12]. Bonferroni-corrected *post hoc* pairwise comparisons revealed greater VAS score on the vividness and emotional arousal subscales in the LSD condition than in the Pla condition and on the vividness subscale in the LSD condition than in the Ket+LSD condition (all p < 0.05). VAS score did not differ between the Pla and Ket+LSD conditions for any VAS subscale (all p = n.s.), indicating that ketanserin pre-treatment completely blocked all LSD-induced effects (**Supplementary Figure S2**).

### Primary and Secondary Process Thinking

Lysergic acid diethylamide significantly increased primary process thinking, as indicated by a significant main effect of drug [*F*(1.07,25.70) = 50.63, *p* < 0.001, $\eta_{p}^{2}$ = 0.6] in a one-way repeated-measures ANOVA on PI; and Bonferroni-corrected *post hoc* comparisons revealing significantly greater PI in the LSD condition than in the Pla and Ket+LSD conditions (all *p* < 0.001). PI did not differ between the Pla and Ket+LSD conditions (*p* = 0.07), indicating that ketanserin pre-treatment completely blocked the effect of LSD on PI (**Table 1**). Furthermore, the LSD-induced increase in PI was driven by an increase in PP, and not by a decrease in SP, as indicated by a significant drug × category (PP, SP) interaction [*F*(1.54,36.87) = 13.30, *p* < 0.001, $\eta_{p}^{2}$ = 0.32] in a separate repeated-measures ANOVA; and Bonferroni-corrected *post hoc* pairwise comparisons revealing greater PP, but unchanged SP, in the LSD condition than in the Pla and Ket+LSD conditions (all *p* < 0.001). PP did not differ between the Pla and Ket+LSD conditions (all *p* = n.s.), indicating that ketanserin pre-treatment completely blocked the effect of LSD on PP (**Table 1**).

**Table 1:** Relative frequencies and *post hoc* pairwise comparisons for primary process, secondary process, and primary index in the three drug conditions.

	Relative frequency^g^			*t_24_* value^h^ *p*-value^i^		
Category	Pla^d^	Ket+LSD^e^	LSD^f^	LSD > Pla	LSD > Ket+LSD	Ket+LSD > Pla
PP^a^	0.0007	0.0012	0.0080	6.40	6.42	1.86
	(0.0013)	(0.0022)	(0.0063)	0.000001^∗∗^	0.000001^∗∗^	0.07
SP^b^	0.0451	0.0459	0.0427	–0.97	–1.80	0.35
	(0.0128)	(0.0105)	(0.0110)	0.34	0.08	0.73
PI^c^	1.3375	2.3237	14.7980	7.26	7.12	2.44
	(1.9622)	(3.2501)	(9.9916)	0.0000002^∗∗^	0.0000002^∗∗^	0.02

*N = 25. ^a^Primary process. ^b^Secondary process. ^c^Primary index, 100 × PP/(PP+SP). ^d^Placebo. ^e^Ketanserin. ^f^Lysergic acid diethylamide. ^g^Mean (SD). ^h^*Post hoc* paired *t* tests, two-tailed. ^i^Uncorrected *p*-value. ^∗^p < 0.05, Bonferroni-corrected; ^∗∗^p < 0.001, Bonferroni-corrected (alpha threshold 0.05/9 = 0.0056).*

*^1^*D*-lysergic acid amide.*

*^2^*N,N*-dimethyltryptamine.*

*^3^2,5-dimethoxy-4-ethylphenethylamine.*

### Relations between Outcome Variables

**FIGURE 1:**
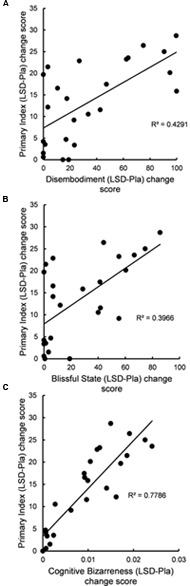
**(A)** Change in disembodiment was related to change in primary index of mental imagery reports. The scatter plot shows the relation between the LSD-induced increase in score on the disembodiment subscale of the 5D-ASC (difference between LSD and placebo drug conditions, *x*-axis) and the LSD-induced increase in primary index score of mental imagery reports (difference between LSD and placebo drug conditions, *y*-axis) (*r* = 0.61, *N* = 25, *p* = 0.012, Bonferroni-corrected); **(B)** Change in blissful state was related to change in primary index of mental imagery reports. The scatter plot shows the relation between the LSD-induced increase in score on blissful state subscale of the 5D-ASC (difference between LSD and placebo drug conditions, *x*-axis) and the LSD-induced increase in primary index score of mental imagery reports (difference between LSD and placebo drug conditions, *y*-axis) (*r* = 0.63, *N* = 25, *p* = 0.012, Bonferroni-corrected); **(C)** Change in cognitive bizarreness was related to change in primary index of mental imagery reports. The scatter plot shows the relation between the LSD-induced increase in cognitive bizarreness score (difference between LSD and placebo drug conditions, *x*-axis) and the LSD-induced increase in primary index score of mental imagery reports (difference between LSD and placebo drug conditions, *y*-axis) (*r* = 0.89, *N* = 25, *p* < 0.001, Bonferroni-corrected). Pla, placebo; LSD, lysergic acid diethylamide.

There was a significant positive correlation between LSD-induced change (LSD-Pla difference score) in PI and LSD-induced change in 5D-ASC scores for the disembodiment subscale (*r* = 0.61, *N* = 25, *p* = 0.012, Bonferroni-corrected) and for the blissful state subscale (*r* = 0.63, *N* = 25, *p* = 0.012, Bonferroni-corrected) (**Figures 1A,B**). Furthermore, there was a highly significant positive correlation between LSD-induced change in PI and LSD-induced change in BD (*r* = 0.89, *N* = 25, *p* < 0.001, Bonferroni-corrected) (**Figure 1C**).

## Discussion

The main finding of this study was that LSD increased primary process thinking, a lower-level, automatic, motivation- and emotion-driven mode of mental organization which is characterized by image fusion; unlikely combinations or events; sudden shifts or transformations of images; and contradictory or illogical actions, feelings, or thoughts (Rapaport, 1950; Holt, 1956; Auld et al., 1968; Shevrin, 1996; Sloman and Steinberg, 1996; Brakel et al., 2000). Specifically, we show that LSD, in comparison with placebo, increased primary index, a formal linguistic measure of primary process thinking in the imagery reports (Auld et al., 1968; Stigler, 2001) (**Table 1**). Furthermore, we found that the effect of LSD on primary index was completely blocked by ketanserin, a 5-HT2A receptor antagonist (**Table 1**). Finally, we show that the LSD-induced increase in primary index was related to LSD-induced disembodiment and blissful state (**Figures 1A,B**).

Our finding that LSD acutely increased primary process thinking is supported by both direct and indirect evidence: Landon and Fischer (1970), for example, assessed the effects of low-dose (0.08 mg/kg orally) psilocybin on several linguistic parameters. It was found that psilocybin decreased sentence length and syntactic and rhetorical complexity, but increased linguistic concreteness and stereotypy, consistent with primary process thinking. Furthermore, Martindale and Fischer (1977) used content-analytic measures to directly test the hypothesis that psilocybin (0.08–0.2 mg/kg) induces primary process thinking. They showed that psilocybin increased primary process words, particularly content related to regressive imagery. Moreover, Natale et al. (1978, 1979) investigated the effects of low to medium dose LSD (15–100 mcg) on speech patterns of depressed patients during psychoanalytic sessions. They found that LSD increased the patients’ use of novel figurative language and of primary process-related words, respectively, consistent with an increase in primary process thinking. Furthermore, Barr et al. (1972) and Holt (2002) investigated the effects of LSD on primary process responses to the Rorschach projective test. They found highly significant LSD effects on formal measures of primary process thinking, including features such as image fusion, fluid transformations of percepts, autistic logic, logical contradictions, verbal condensations, loosening of memory, and unlikely combinations. Finally, recent double-blind, placebo-controlled studies lend further support to the notion that psychedelics enhance primary process thinking: Spitzer et al. (1996) for example, used word-pairs of different semantic distance and showed that psilocybin increased indirect semantic priming, i.e., priming for remotely related word-pairs. Interestingly, the authors interpreted their results as evidence that psilocybin “in fact leads to an increased availability of remote associations and thereby may bring cognitive contents to mind that under normal circumstances remain non-activated.” Similar effects were found for LSD in a recent double-blind, placebo-controlled study by Family et al. (2016). Taken together, both our results and previous evidence indicate that psychedelics induce an altered state of consciousness which is characterized by primary process cognition. Our findings are also in line with recent neuroimaging data: the entropic brain theory (Carhart-Harris et al., 2014), for example, holds that secondary process (the cognitive mode of the Freudian “ego”) is coded by default mode network (DMN) regions and provides top–down predictions to reduce free-energy associated with the primary process (the Freudian “id”) within (para)limbic and anti-correlated neural networks, converting free energy into bound energy. According to the entropic brain theory, psychedelics induce an “unconstrained,” “high-entropy” cognitive state whereby DMN activity breaks down, leading to broadband alterations in resting-state functional connectivity between regions that show little connectivity in a baseline state.

However, contrary to such cognitive shift models, which posit that psychedelics decrease secondary process thinking, leading to disinhibition of primary process thinking (“ego regression,” in psychoanalytical terms), our data did not show a statistically significant effect of LSD on SP, while there was a significant increase in PP during LSD compared to placebo (**Table 1**). These findings seem to suggest that there is no simple “shift” or “transition” from secondary toward primary process thinking during psychedelic states: secondary process thinking during psychedelic states appears preserved, while there is an increase in primary process thinking. This may be an important feature distinguishing night dreams from psychedelic experiences. In fact, a recent neuroimaging study (Lewis et al., 2017) showed that psychedelics increase rather than decrease neural activity in cortical areas that are thought to mediate the features of secondary process thinking, including dorsolateral prefrontal cortex (DLPFC) and temporal cortex (Dresler et al., 2014). Therefore, psychedelic states may be best conceptualized as hybrid states of consciousness which share features of both dreaming and waking consciousness. This is supported by a recent neuroimaging study (Voss et al., 2014) which showed that dreaming (and hence unaware) subjects regained self-awareness in their dreams (they became “lucid”) following frontal low current stimulation of gamma activity over DLPFC regions. In fact, the close neurophenomenological similarity between psychedelic states and lucid dreaming (Kraehenmann, 2017) may shed some light on the therapeutic potential of psychedelic-induced experiences: they are not just “epiphenomena” of underlying neuronal oscillations, but rather induce conscious learning experiences that promote self-knowledge and psychological insight.

The human brain is a hierarchically organized and evolutionally layered organ, and this basic structure is reflected in the cognitive organization of the mind (Montag and Panksepp, 2017). Primary process thinking has been related to neuronal activation of ontologically early, subcortical and limbic regions of the brain which code for instinctual drives and primal affective experiences (Solms and Panksepp, 2012; Montag and Panksepp, 2017). In addition, previous neuroimaging studies (Carhart-Harris et al., 2012; Kraehenmann et al., 2015, 2016; Lewis et al., 2017) indicate that psychedelics such as psilocybin modulate information processing in both cortical and subcortical memory and emotion circuits of the brain (e.g., cingulate cortex, temporal cortex, insula, amygdala, hippocampus). This is supported by recent receptor binding studies showing a dense and widespread expression of 5-HT2A/5-HT1A receptors in both cortical and subcortical areas of the human brain (Beliveau et al., 2017). This may explain why, in psychedelic states, basic drives and primary emotions are strongly activated and substantially influence cognition and behavior (Hermle and Kraehenmann, 2017). In fact, there is consistent evidence that psychedelics, especially during drug peak effects, induce high emotional arousal: “…intense, labile, personally meaningful emotionality is uniformly produced, with periodic episodes of overwhelming feeling” (Pahnke et al., 1971). Even under high-dose drug conditions, most subjects describe their imagery as highly pleasurable and rewarding (“cosmic joy”), coming along with feelings of “boundlessness” and “unity” (“oceanic boundlessness”) (Griffiths et al., 2011; Studerus et al., 2011). Moreover, previous factor analytical studies (Studerus et al., 2010, 2011; Lebedev et al., 2015) support the view that psychedelics induce altered states of consciousness based on two main factors: visual imagery on the one hand, and emotionally experienced alterations in self-awareness and loss of self-/body-boundaries on the other hand. Taken together, our results are entirely consistent with this view because LSD significantly induced vivid imagery on the VAS subscale, blissful state (positively valenced mood state) and disembodiment on the 5D-ASC subscale (**Supplementary Figure S1**).

Recent behavioral (Kometer et al., 2012, 2013; Kraehenmann et al., 2017) and neurobiological studies (Lebedev et al., 2015; Preller et al., 2017) may help explain why psychedelics are such potent modulators of visual imagery, emotions, and self-/body-awareness. For example, it has been shown that 5-HT2A receptor activation in the brain is a central mechanism underlying psychedelic-induced imagery (Kometer et al., 2013), positive mood states (Kometer et al., 2012), and alterations in the sense of self and body (Vollenweider et al., 1998). Therefore, our results are consistent with this view because ketanserin-pretreatment of LSD completely blocked the observed subjective and behavioral effects of LSD (**Table 1** and **Supplementary Figure S1**). Given that 5-HT receptors are widely expressed in the human brain (Beliveau et al., 2017), they have important functions in the regulation of mood states, instinctual drives, sleep, and dreaming (Nichols and Nichols, 2008; Pace-Schott, 2008). In fact, we (Kraehenmann et al., 2017) have recently shown that 5-HT2A receptor activation by LSD induces dreamlike imagery, correlating with LSD-induced loss of self-boundaries and cognitive control. Given that there is a broad phenomenological and neurophysiological overlap between psychedelic states and dreaming (Kraehenmann, 2017), and given that primary process thinking is the prevalent cognitive mode in dreams (Rapaport, 1950; Holt, 1956; Auld et al., 1968; Shevrin, 1996; Sloman and Steinberg, 1996; Brakel et al., 2000), it is plausible to assume that 5-HT2A receptor activation by psychedelics induces dreamlike imagery which is related to primary process thinking, emotion activation, and alterations in the sense of self and body. This is strongly supported by our results because LSD-induced primary process thinking was positively correlated with LSD-induced cognitive bizarreness, a formal measure of dreaming cognition (**Figure 1C**), and was related to both LSD-induced blissful state (**Figure 1B**) and disembodiment (**Figure 1A**) on the 5D-ASC. Finally, this is also supported by previous neuroimaging studies (Maquet et al., 1996; Braun, 1997; Solms, 2000; Lebedev et al., 2015) which found that both psychedelics and rapid-eye movement dreams activate temporal lobe regions, leading to visual imagery and changes in the sense of self and body.

The close relationship between primary process thinking, dream-like cognitive bizarreness, imagery intensity and emotionality during LSD in conjunction with guided mental imagery relative to guided imagery during placebo implicates that LSD in combination with mental imagery induces inner experiences which are different from those produced by either LSD alone or guided mental imagery alone. Given that mental imagery and dreams establish privileged access to latent relational and emotional schemes (Grenell, 2008; Kottje-Birnbacher, 2011), LSD and other classical psychedelics might be beneficially used as add-on pharmacotherapeutics to deepen psychotherapeutic processes (Kraehenmann, 2017). In fact, early clinical studies between 1950 and 1970 used LSD in a similar way (Strassman, 1995). Levine and Ludwig (1965), for example, showed that the combination of hypnosis and LSD produced more profound alterations in consciousness than either hypnosis or LSD alone. Future clinical studies could test this hypothesis by using a study design with several treatment arms comparing either psychedelics without psychotherapy versus psychedelics in conjunction with psychotherapy versus psychotherapy alone.

### Limitations

We only assessed primary process thinking during the descending phase of the acute effects of LSD (Dolder et al., 2015). Therefore, we didn’t measure drug peak-effects, which might have yielded different results. Nonetheless, we are confident that peak-effects of LSD on cognition and subjective experience would turn out to be similar, if not even stronger, than the observed effects, given that during drug peak, the effects of LSD were completely blocked by ketanserin, and given that LSD induced more primary process in subjects which had more intense subjective drug effects (**Figures 1A,B**). However, we did not assess dose-dependency of the effects of LSD on primary process thinking. Given that a recent neuroimaging study (Lewis et al., 2017) did not find dose-dependent differences between brain activation patterns in the acute psychedelic state, and given that dose-response relationships for psychedelic drug effects are approximately linear (Studerus et al., 2011), we expect the effects of LSD on primary process thinking to linearly increase with increasing dose.

## Conclusion

We found that LSD, compared with placebo, enhanced primary process thinking in relation to disembodiment and blissful state. Our results confirm previous studies which showed that psychedelics acutely increase primary process thinking. Furthermore, our results indicate that psychedelic-induced primary process thinking is closely related to 5-HT2A receptor activation and the effects on mood state and sense of self and body. Taken together, we show that psychedelics induce transient, but fundamental changes in consciousness which otherwise are only experienced under psychophysiological conditions where primary process is activated such as in dreams. Finally, the results of this study may help extend current understanding of the cognitive mechanisms underlying psychedelic-induced subjective experience. Future clinical studies may test the hypothesis that therapeutic efficacy is mediated by the psychedelic-induced primary process thinking.

## Author Contributions

Each of the authors participated in this research by contributing to the conception and design of the study (RK, DP, KP, and FV), study management (RK, KP, TP, ES, and FV) performance of laboratory experiments (RK, KP, and TP) and statistical analysis and interpretation (RK, DP, HA, OB, ES, and FV).

## Conflict of Interest Statement

The authors declare that the research was conducted in the absence of any commercial or financial relationships that could be construed as a potential conflict of interest.

## Supplementary Material

FIGURE S1 State of consciousness at the time of the mental imagery task. The graph shows the score on each 5D-ASC subscale in each drug condition at time point T3 = 390 min after drug intake. LSD increased the score on all five 5D-ASC subscales. Ketanserin pre-treatment completely blocked all LSD-induced effects (all *p* = n.s.). Asterisks indicate significant differences between LSD and placebo conditions (^∗^*p* < 0.05; ^∗∗^*p* < 0.001, Bonferroni-corrected). 5D-ASC, Altered States of Consciousness; Pla, placebo; Ket, ketanserin; LSD, lysergic acid diethylamide.

FIGURE S2 Mental imagery experience. The graph shows the score on each visual analog subscale (VAS) in each drug condition. LSD increased VAS score for visual vividness and emotional arousal. Ketanserin pre-treatment completely blocked all LSD-induced effects (all *p* = n.s.). Asterisks indicate significant differences between LSD and placebo conditions (^∗^*p* < 0.05; ^∗∗^*p* < 0.001, Bonferroni-corrected). Pla, placebo; Ket, ketanserin; LSD, lysergic acid diethylamide.
