# Supplementary figures and images for: LSD Increases Primary Process Thinking via Serotonin 2A Receptor Activation
Source: Front Pharmacol. 2017 Nov 8;8:814. doi: 10.3389/fphar.2017.00814 (PMC5682333; doi:10.3389/fphar.2017.00814)

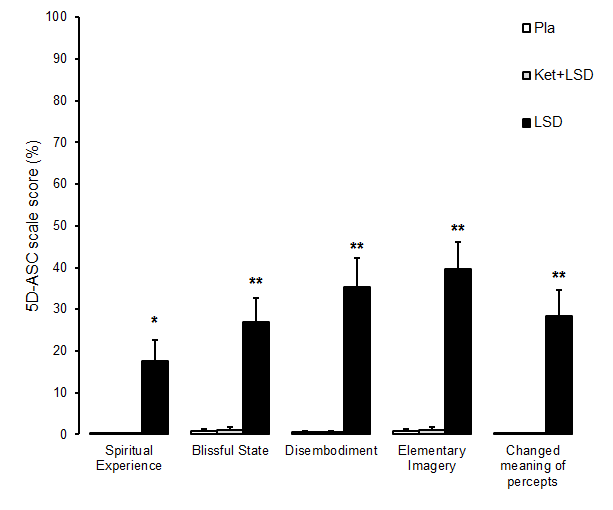

Supplement: FIGURE S1 — State of consciousness at the time of the mental imagery task. The graph shows the score on each 5D-ASC subscale in each drug condition at time point T3 = 390 min after drug intake. LSD increased the score on all five 5D-ASC subscales. Ketanserin pre-treatment completely blocked all LSD-induced effects (all p = n.s.). Asterisks indicate significant differences between LSD and placebo conditions (∗p < 0.05; ∗∗p < 0.001, Bonferroni-corrected). 5D-ASC, Altered States of Consciousness; Pla, placebo; Ket, ketanserin; LSD, lysergic acid diethylamide. [file Image_1.tif]
